# Use of Amphoteric Copolymer Films as Sacrificial Layers for Constructing Free-Standing Layer-by-Layer Films

**DOI:** 10.3390/ma6062351

**Published:** 2013-06-06

**Authors:** Baozhen Wang, Yu Tokuda, Koji Tomida, Shigehiro Takahashi, Katsuhiko Sato, Jun-ichi Anzai

**Affiliations:** 1 Department of Nutrition and Food Hygiene, School of Public Health, Shandong University, 44 Wenhua Xilu, Jinan, Shandong 250012, China; E-Mail: bzhenw@hotmail.com; 2 Graduate School of Pharmaceutical Sciences, Tohoku University, Aramaki, Aoba-ku, Sendai 980-8578, Japan; E-Mails: b0ym1020@s.tohoku.ac.jp (Y.T.); a8yb1058@s.tohoku.ac.jp (K.T.); t-shigehiro@m.tohoku.ac.jp (S.T.); satok@m.tohoku.ac.jp (K.S.)

**Keywords:** layer-by-layer film, amphoteric copolymer, free-standing LbL film, sacrificial layer, poly(diallylamine-*co*-maleic acid)

## Abstract

The present paper reports the use of an amphoteric copolymer, poly(diallylamine-*co*-maleic acid) (PDAMA), as a component of precursor layers (or sacrificial layers) for constructing free-standing layer-by-layer (LbL) films. A PDAMA-poly(styrenesulfonate) (PSS) film or PDAMA-poly(dimethyldiallylammonium chloride) (PDDA) film was coated on the surface of a quartz slide at pH 4.0 or 8.0, respectively, as a sacrificial layer that can be removed by changing the pH. The surface of the sacrificial layer was further covered with LbL films composed of poly(allylamine hydrochloride) (PAH) and PSS. The PAH-PSS films were released from the substrate upon immersing the film-coated quartz slide in acidic or neutral/basic solution, respectively, as a result of the pH-induced dissolution of the PDAMA-PDDA or PDAMA-PSS sacrificial layer. Thus, PDAMA-based sacrificial layers have been demonstrated to dissolve in both acidic and neutral solutions, depending on the type of counter polymer. The thicknesses of the sacrificial layers and released LbL films are crucial factors for constructing free-standing LbL films. The releasing kinetics also depended on the thickness of the crucial layers. The free-standing PAH-PSS films obtained were stable in water or in air in the dry state. PDAMA-based sacrificial layers may be useful in constructing free-standing LbL films containing biomolecules with limited pH stability.

## 1. Introduction

The layer-by-layer (LbL) deposition technique has attracted much attention as a bottom-up nanofabrication process for preparing microcapsules and nanoscale thin films, because of its potential applications in surface modification [1,2], separation and purification membranes [3], molecular architectures [4], electronic and optical devices [5,6], stimuli-sensitive systems [7,8], drug delivery [9,10,11], and so forth. A variety of materials, such as synthetic polymers [12], proteins [13], polysaccharides [14,15] and dendrimers [16], have been employed as building blocks of LbL films. Recently, free-standing LbL films have been prepared by releasing them from the surface of solid substrates [17,18,19,20,21,22,23]. In this procedure, the surface of the substrate is first covered with so-called sacrificial layers, which dissolve in solutions in response to external stimuli, such as temperature [17,18], specific ions [19], salts [20] and pH changes [21,22,23]. Among stimuli-sensitive materials, pH-sensitive LbL films whose solubility is pH-dependent have often been used as sacrificial layers for this purpose. For instance, free-standing films composed of poly(allylamine hydrochloride) (PAH) and poly(styrenesulfonate) (PSS) have been prepared by using hydrogen-bonded LbL films made of poly(acrylic acid) (PAA) and poly(ethylene glycol) (PEG) as sacrificial layers [24]. A PAA-PEG-layer-coated silicon wafer was further coated with a PAH-PSS film at pH 3.0, and the PAA-PEG layer was dissolved in neutral solutions to release the PAH-PSS film from the substrate. The pH-dependent dissolution of the PAA-PEG layer was ascribed to the breakage of hydrogen bonds as a result of the deprotonation of PAA. In another study, electrostatically bonded LbL film composed of poly(dimethyldiallylammonium chloride) (PDDA) and zwitterionic poly(4-vinylpyridine propylsulfonate) (PVPPS) was employed as a sacrificial layer, by which free-standing films were released at pH 12 [25]. It was also possible to prepare free-standing PAA-PAH films at pH 3.6 in the presence of Cu^2+^ [19]. These studies show that free-standing LbL films can be constructed in a limited pH range, depending on the pH stability of the sacrificial layers. Therefore, it would be valuable if the pH stability of sacrificial layers could be arbitrarily controlled.

**Figure 1:**
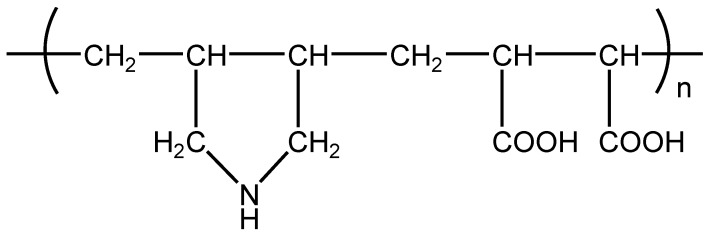
Chemical structure of poly(diallylamine-*co*-maleic acid) (PDAMA).

Toward this end, we have used here an amphoteric copolymer, poly(diallylamine-*co*-maleic acid) (PDAMA) (Figure 1), as a component of sacrificial layers for constructing free-standing LbL films. PDAMA-based LbL films can be decomposed at both acidic and neutral/basic pHs, depending on the counter polymer, owing to the amphoteric nature of PDAMA [26]. That is, LbL films composed of PDAMA and anionic polymers, which were prepared at acidic pH, can be decomposed in neutral or basic solutions, because the net charge of PDAMA shifts from positive to negative in neutral/basic solutions. Similarly, PDAMA-polycation films prepared at basic pH can be decomposed and dissolved in acidic solutions. Consequently, free-standing LbL films may be prepared using PDAMA-based sacrificial layers at both acidic and neutral/basic pHs. To our knowledge, no amphoteric polymer has been employed to construct free-standing LbL films.

## 2. Experimental Section

PSS (MW, ~500,000) and an aqueous solution (20%) of PDDA (MW, 100,000–200,000) were obtained from Aldrich Co. (Milwaukee, WI, USA). PAH (MW, ~10,000) was purchased from Nitto Bouseki Co. Ltd. (Tokyo, Japan). PDAMA hydrochloride was kindly donated by Nitto Bouseki Co. Ltd. PDAMA is a copolymer that consists of alternating diallylamine and maleic acid monomer units [26]. The deposition behavior of the LbL films was gravimetrically evaluated with a quartz crystal microbalance (QCM; QCA 917, Seiko EG & G, Tokyo, Japan). A 9 MHz AT-cut quartz resonator coated with a thin Au layer (0.2 cm^2^) was used as the probe, in which the adsorption of 1 ng of substance induced a −0.91 Hz change in resonance frequency. The Au-coated probe was immersed in 5 mM 3-mercaptopropanesulfonate aqueous solution overnight to make the surface negatively charged. LbL films composed of (PDDA-PDAMA)*_m_* layers and (PAH-PSS)*_n_* layers were successively deposited on both surfaces of the quartz resonator by alternately immersing the probe in polymer solutions for 5 min, followed by 30 s of rinsing in buffer. The polymer solutions used were 0.6 mg/mL PDAMA, 1 mg/mL PDDA, 1 mg/mL PAH and 1 mg/mL PSS solutions prepared in 10 mM Tris-HCl buffer (pH 8.0). The change in the resonance frequency (∆F) of the quartz resonator was recorded in the dry state after the deposition of each polymer and rinsing. LbL films composed of PSS-PDAMA sacrificial layers and PSS-PAH layers were also prepared in a similar manner using 10 mM acetate buffer at pH 4.0. For spectroscopic evaluation of film deposition, the LbL films were prepared on the surface of a quartz slide and absorption spectra were recorded with a UV-visible absorption spectrometer (UV-3100PC, Shimadzu, Kyoto, Japan). The LbL film-coated glass slides were gently shaken in acidic or neutral/basic buffer solution to release the (PAH-PSS)*_n_* or (PSS-PAH)*_n_* film from the substrate.

## 3. Results and Discussion

**Figure 2:**
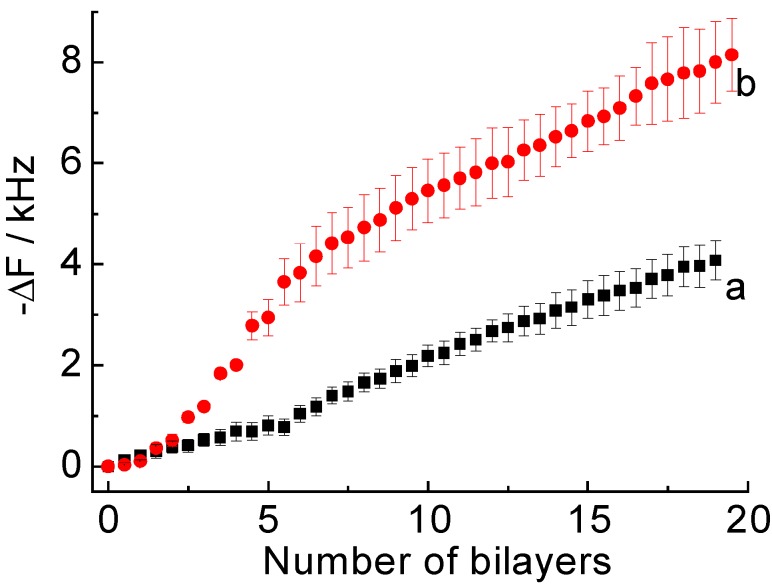
Frequency changes in the quartz crystal microbalance (QCM) for the deposition of (**a**) PDAMA(PDDA-PDAMA)_5_ + (PAH-PSS)_14_ film at pH 8.0 and (**b**) PDDA(PSS-PDAMA)_5_ + (PSS-PAH)_14_ film at pH 4.0. The averages of three independent measurements are plotted.

Figure 2 shows the frequency changes in the QCM upon depositing PDDA(PSS-PDAMA)_5_ + (PSS-PAH)_14_ and (PDDA-PDAMA)_5_ + (PAH-PSS)_14_ films in acidic and neutral solutions, respectively. In both cases, ∆F decreased with increasing number of layers, confirming the successful deposition of LbL films under the experimental conditions. The results imply that PDAMA is positively charged at pH 4.0, while negatively charged at pH 8.0, owing to the acid-base equilibrium of the diallylamine and maleic acid moieties. The thickness of the (PDDA-PDAMA)_5_ layers linearly increased as the number of layers was increased. However, the thickness increase for the PDDA(PSS-PDAMA)_5_ layers in the first few layers was smaller than that observed in the following layers. The thicknesses of the PDDA(PSS-PDAMA)_5_ and (PDDA-PDAMA)_5_ layers were estimated from the QCM data to be ~85 ± 10 nm and ~20 ± 4 nm, respectively, assuming that the density of the polymer films was 1.2 g/cm^3^ [27]. On the other hand, the thicknesses of the (PSS-PAH)_14_ and (PAH-PSS)_14_ films coated on the PDDA(PSS-PDAMA)_5_ and (PDDA-PDAMA)_5_ layers were calculated to be ~100 ± 15 nm and ~80 ± 10 nm, respectively.

**Figure 3:**
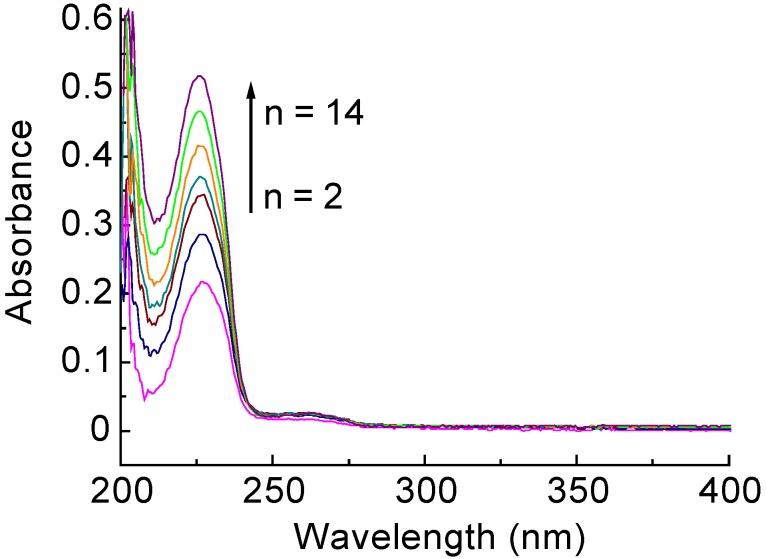
Typical UV-visible absorption spectra of PDDA(PSS-PDAMA)_5_+(PSS-PAH)*_n_* films (*n =* 2, 4, 6, 8, 10, 12 and 14).

The deposition behavior of the LbL films was further studied by UV-visible absorption spectrometry. The LbL films were deposited on the surface of a quartz slide, which had been cleaned by using a mixture of chromic acid and sulfuric acid. Figure 3 shows typical absorption spectra of the PDDA(PSS-PDAMA)_5 _+ (PSS-PAH)_14_ films, which were prepared in a similar manner to the films deposited on the QCM quartz resonator. The LbL films exhibited a clear absorption band at 225 nm, originating from the aromatic ring in PSS. The intensity of the absorption increased with the increasing number of layers, indicating the successful deposition of the LbL film.

**Figure 4:**
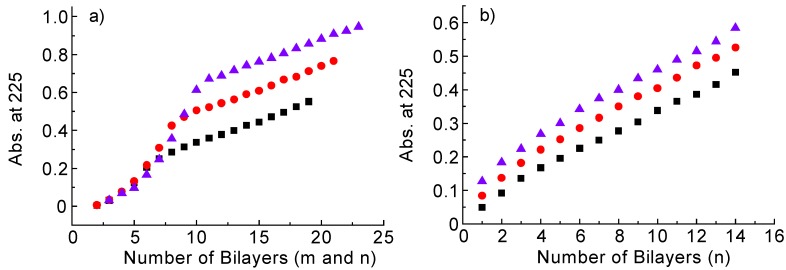
(**a**) Absorbance of PDDA(PSS-PDAMA)*_m _* + (PSS-PAH)*_n_* films as a function of the number of layers (■, *m =* 5; ●, *m =* 7; and ▲, *m =* 9). The final number of the (PSS-PAH)*_n_* layers was 14 for all the films; (**b**) absorbance of (PDDA-PDAMA)*_m_*+(PAH-PSS)*_n_* films as a function of the number of (PAH-PSS)*_n_* layers (■, *m =* 3; ●, *m =* 5; and ▲, *m =* 7). The plots for the absorbance of the (PDDA-PDAMA)*_m_* layers are omitted for clarity.

Figure 4a shows plots of the absorbance of the PDDA(PSS-PDAMA)*_m_* + (PSS-PAH)_14_ films (*m =* 5, 7 and 9) at 225 nm as a function of the number of bilayers. These results show that (PSS-PAH)_14_ films can be prepared on PDDA(PSS-PDAMA)*_m_* layers, irrespective of the thickness of the sacrificial layers. The slopes of the plots for the (PAH-PSS)_14_ film depositions are nearly identical to each other, showing that the thickness of (PSS-PAH)_14_ films was independent of sacrificial layer thickness. Figure 4b shows plots of the results for the (PAH-PSS)_14_ films deposited on the surface of (PDDA-PDAMA)*_m_* layers (*m* = 3, 5 and 7). In this figure, the plots of the absorbance of the (PDDA-PDAMA)*_m_* layers are omitted for clarity, because PDDA and PDAMA contain no distinct absorption band in the spectral region. The absorbance of the (PAH-PSS)_14_ films linearly increased with an increasing number of layers, confirming the successful deposition of the (PAH-PSS)_14_ films on the (PDDA-PDAMA)*_m_* layers. Thus, both the gravimetric and spectroscopic data support the preparation of LbL films composed of PDAMA-based sacrificial layers and PAH-PSS layers.

It is reasonable to assume that the sign of the net electric charge of PDAMA depends on the pH of the medium, because diallylamine and maleic acid moieties in PDAMA are dissociable. PDAMA should have a positive net charge in acidic solutions, but a negative net charge in neutral and basic media. These considerations suggest that the PDDA(PSS-PDAMA)*_m_* segment in the PDDA(PSS-PDAMA)*_m _*+ (PSS-PAH)_14_ films, which were prepared in acidic solution, can be decomposed in neutral/basic solutions, because PDAMA acquires a negative net charge, thereby losing its electrostatic affinity to PSS. In other words, (PSS-PAH)_14_ films are released from the substrate surface as a result of the decomposition of the PDDA(PSS-PDAMA)*_m_* inner layers. In fact, we have found that (PSS-PAH)_14_ films are released from the substrate surface upon gently shaking the PDDA(PSS-PDAMA)_5 _+ (PSS-PAH)_14_-film-coated substrate in a weak basic solution (10 mM Tris-HCl buffer, pH 8.0) at room temperature [28]. Similarly, the immersion of the (PDDA-PDAMA)_5_ + (PAH-PSS)_14_ films in acidic solution (dilute HCl, pH 2.0) afforded free-standing LbL films through the decomposition of the (PDDA-PDAMA)_5_ segment as a result of the charge shift of PDAMA from negative to positive. These results show that PDAMA-based LbL layers can be used as sacrificial layers in both acidic and neutral solutions for constructing free-standing LbL films.

**Figure 5:**
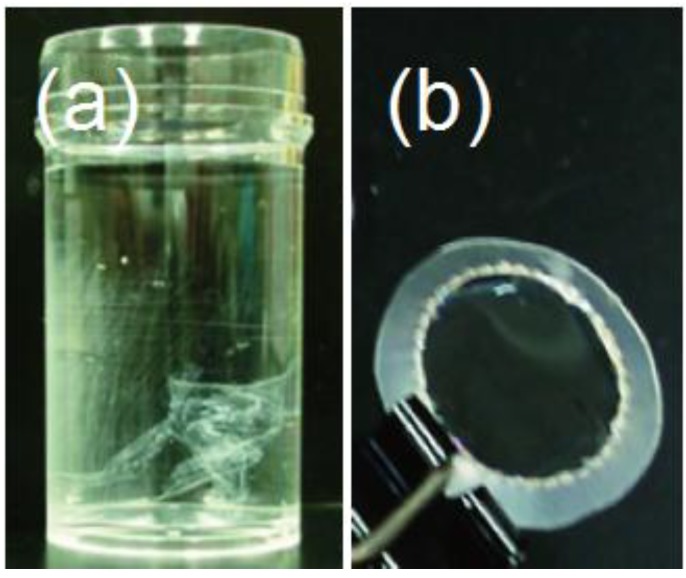
Photographs of typical free-standing film (**a**) in Tris-HCl buffer at pH 8.0 and (**b**) in the dry state. The films were released from PDDA(PSS-PDAMA)_5 _+ (PSS-PAH)_11_PSS film.

The thicknesses of the sacrificial layers, as well as of the released LbL films may be crucial factors for preparing free-standing LbL films. Therefore, the effects of the thickness of the sacrificial layers were evaluated using PDDA(PSS-PDAMA)*_m _*+ (PSS-PAH)_14_ and (PDDA-PDAMA)*_m _*+ (PAH-PSS)_14_ films (*m =* 1, 2, 3, 4, 5 and 7). For the PDDA(PSS-PDAMA)*_m _*+ (PSS-PAH)_14_ films, sacrificial layers with five bilayers or more were required for the release of free-standing (PSS-PAH)_14_ films at pH 8.0. In contrast, for the (PDDA-PDAMA)*_m _*+ (PAH-PSS)_14_ films, a three-bilayer (PDDA-PDAMA)_3_ film sufficed to release the (PAH-PSS)_14_ film at pH 2.0. Thinner sacrificial films were ineffective in releasing the free-standing films, probably because the (PAH-PSS)_14_ and (PSS-PAH)_14_ films were weakly bound directly to the substrate surface, as a result of the partial penetration of the PAH or PSS chains into the thinner sacrificial layers. It is known that polymer chains often interpenetrate into adjacent layers in LbL films [18,24,29]. The (PSS-PAH)_14_ film was released from the PDDA(PSS-PDAMA)*_m_* + (PSS-PAH)_14_ films in 4–7 min in the pH range 8.0–10.0. On the other hand, the film release from the (PDDA-PDAMA)*_m _*+ (PAH-PSS)_14_-film-coated slide was rather slow and thickness-dependent; it took 30, 45 and 50–60 min to release the (PAH-PSS)_14_ film from the (PDDA-PDAMA)_5_, (PDDA-PDAMA)_4_ and (PDDA-PDAMA)_3_ sacrificial layers, respectively. The release of the (PAH-PSS)_14_ film was faster when the sacrificial layer was thicker. A similar trend in the kinetics of the thickness-dependent release of free-standing LbL films has been reported for temperature-sensitive sacrificial layers [18]. The critical thickness of the free-standing (PAH-PSS)*_n_* and (PSS-PAH)*_n_* films was found to be four or five bilayers, which corresponds to 25–35 nm in thickness. Figure 5 shows photographs of typical free-standing films. The free-standing film was stable in water for a few months without deterioration (Figure 5a). LbL films thicker than the critical value could be taken out of the water and dried in air (Figure 5b). The dried LbL films were nearly transparent and stable in air. In contrast, thinner films decomposed into fragments when the sacrificial layer was dissolved in water. Thus, the thicknesses of the sacrificial layers and released films are crucial factors in preparing free-standing LbL films. In this context, the critical thickness of poly(acrylic acid)-based sacrificial layers has been reported to be ~117 nm [24].

## 4. Conclusions

PDAMA-PDDA and PDAMA-PSS films dissolve in acidic and neutral/basic solutions, respectively, owing to the amphoteric nature of PDAMA. Thus, PDAMA-based LbL films can be used as sacrificial layers for preparing free-standing LbL films in both acidic and neutral/basic media. The present results may be useful in the construction of free-standing LbL films containing biomolecules, such as proteins, with limited pH stability.
